# Retraction Note: Newcastle disease virus enhances the growth-inhibiting and proapoptotic effects of temozolomide on glioblastoma cells in vitro and in vivo

**DOI:** 10.1038/s41598-026-36521-2

**Published:** 2026-01-19

**Authors:** Yang Bai, Yong Chen, Xinyu Hong, Xinrui Liu, Xing Su, Shanji Li, Xuechao Dong, Gang Zhao, Yunqian Li

**Affiliations:** 1 Department of Neurosurgery, First Hospital of Jilin University, Changchun, China; 2 Laboratory of Cancer Precision Medicine, First Hospital of Jilin University, Changchun, China

Retraction of: *Scientific Reports* 10.1038/s41598-018-29929-y, published online 31 July 2018

The Editors have retracted this Article.

After publication, concerns were raised about some of the data in this Article. Specifically:

- the second panel of the first column of Fig. 6 appears to partially overlap with the first panel of the first column of Supplementary Fig. 4B in another paper [1] (-2° rotation of the latter subimage).
- the third panel of the first column of Fig. 6 appears to partially overlap with the first panel of the second column of Supplementary Fig. 4B of [1] (-1° rotation of the latter subimage).

The Authors did not respond to the concerns raised. The Editors have therefore lost confidence in the data and conclusions of this Article.

Yang Bai, Yong Chen, Xinyu Hong, Xinrui Liu, Xing Su, Shanji Li, Xuechao Dong, Gang Zhao, and Yunqian Li did not respond to correspondence from the Publisher about this retraction.
